# Get to the point in intensive care medicine - the sooner the better?

**DOI:** 10.1186/cc11506

**Published:** 2013-03-12

**Authors:** Martin Westphal

**Affiliations:** 1 Fresenius Kabi AG, Else-Kröner-Straße 1, 61352 Bad Homburg, Germany; 2 Department of Anesthesiology, Intensive Care and Pain Medicine, University Hospital of Muenster, Albert-Schweitzer-Campus 1, Building A1, 48149 Muenster, Germany

## Abstract

Timing of therapy plays a pivotal role in intensive care patients. Although being evident and self-explanatory, it has to be considered that the appropriateness of a specific therapeutic intervention is likewise important. In view of antibiotic therapy of critically ill patients, the available evidence supports the concept of hitting hard, early (as soon as possible and at least before the onset of shock) and appropriately. There is increasing evidence that a positive fluid balance is not only a cosmetic problem but is associated with increased morbidity. However, prospective studies are needed to elucidate whether a positive net fluid balance represents the cause or the effect of a specific disease. Since central venous pressure (CVP) is an unreliable marker of fluid responsiveness, its clinical use to guide fluid therapy is questionable. Dynamic hemodynamic parameters seem to be superior to CVP in predicting fluid responsiveness in hemodynamically unstable patients. Sedation is often used to facilitate mechanical ventilation. Since there is no best evidence-based sedation protocol, weaning strategies should take the risk of iatrogenic arterial hypotension secondary to high doses of vasodilatory sedative agents into account. In this regard, the concept of daily wake-up calls should be challenged, because higher cumulative doses of sedatives may be required. The right dose and timing for renal replacement therapy is still discussed controversially and remains a subjective decision of the attending physician. New renal biomarkers may perhaps be helpful to validate when (and how) renal replacement therapy should be performed best. Last but not least, all therapeutic interventions should take the individual co-morbidities and underlying pathophysiological conditions into account.

## Introduction

Discussing the meaning of time in the context of intensive care medicine implies that one has to deal with one of the oldest questions of mankind; that is, 'what is time?' The answer can be elaborated philosophically ('Time ... is what keeps everything from happening at once'; Ray Cummings, 1922), economically ('Time is money'; Benjamin Franklin, 1748), or physically ('Time is relative'; Albert Einstein, 1905).

Modern medicine - and especially intensive care medicine - is currently more time-dependent than ever; not only because of the increasing importance of economic aspects, but also due to the meaning of early organ support in the perspective of patient-centered outcome. However, one should critically pose the question of whether it always makes sense to equate the meaning of 'time-saving' with 'better'. A critical view on some well-established concepts (dogmas) therefore appears to be timely and may also help in evaluating whether the views of Cummings, Franklin and Einstein are transferable to modern intensive care medicine.

## Antibiotic therapy in septic patients

At first glance, one may assume that there is no controversy concerning the timing of antibiotic administration in septic patients. According to the Surviving Sepsis Campaign Guidelines 2008, antibiotic therapy should be initiated 'as early as possible and always within the first hour of recognizing severe sepsis (1D) and septic shock (1B)' [1]. Furthermore, the well-known Tarragona strategy implies one should 'hit hard and early' with antibiotic therapy in septic patients [2]. If this concept is commonly accepted, why is it important to think again about the right timing of antibiotic therapy?

In this context, it is noteworthy that Kumar and colleagues performed a retrospective cohort study with 2,731 septic shock patients. The primary endpoint of this trial was to determine the impact of antibiotic timing on survival to hospital discharge. In fact, the investigators reported a strong correlation between delay in effective antibiotic therapy and in-hospital mortality after recurrent or persistent arterial hypotension (*P *<0.0001) [3]. In addition, Kumar and colleagues noticed a decrease in survival by 7.6% for the delay of 1 hour of antibiotic therapy over the ensuing 6 hours. The authors concluded that survival is significantly improved following effective antimicrobial therapy within the first hour after the onset of arterial hypotension. Antibiotic therapy was considered effective, when there was appropriate *in vitro *activity for the isolated pathogenic microorganism or the underlying clinical syndrome. In this context it is especially important to note that only 50% of the patients received effective antibiotic therapy within the first 6 hours [3]. Although the observation of an hourly increase of mortality by 7.6% appears to be pretty high (extrapolated death of 100% after a delay of 13.2 hours), the strong association between initiation of early antibiotic therapy in septic patients with arterial hypotension and survival represents a meaningful (and at the same time logical) finding.

A single-center cohort study by Gaieski and colleagues in 261 patients with severe sepsis undergoing early goal-directed therapy (EGDT) examined the effects of time from triage and from qualification for EGDT to antibiotic administration, as well as the meaning of appropriateness, on survival. At first glance it appears surprising that the authors noticed no significant correlation between mortality and time from triage or qualification for EGDT to antibiotics at different hourly cutoff points [4]. However, time from triage and qualification for EGDT to appropriate antibiotic therapy was significantly associated with reduced mortality at the <1 hour cutoff point (odds ratio = 0.3 and 0.5, each *P *<0.03). The authors concluded that the delay to administration of appropriate antimicrobial therapy represents the primary determinant of mortality in septic shock patients [4].

Puskarich and colleagues performed a multicenter controlled trial in US emergency departments and enrolled 291 patients with septic shock. Addressing the time point of initial antibiotic administration, the patients were categorized into time from triage and into time from shock recognition to antibiotics [5]. Interestingly, the authors found no change in mortality with hourly delayed antibiotic therapy up to 6 hours after triage and after recognition of shock. *Vice versa*, antibiotic administration before recognition of shock was associated with a lower mortality as compared with antibiotic administration after recognition of shock (odds ratio = 2.35, 95% confidence interval = 1.12 to 4.53) [5].

When summarizing the above-referenced studies, it becomes obvious that time plays a crucial factor in antibiotic treatment of patients with sepsis and septic shock. Since self-healing of a severe systemic inflammation is unlikely, common sense indicates that one should initiate antibiotic therapy as soon as possible. However, it should be taken into account that it is not enough to hit hard and early alone. While many therapeutic strategies focus on the role of time, the meaning of an effective and appropriate antibiotic therapy also has to be considered. In view of the Surviving Sepsis Campaign Guidelines and the Tarragona strategy, it makes sense to 'hit hard, early *and *appropriately'. Since trying to get to the point without a clear target makes little sense, the antibiotic weapons should be chosen wisely to make the first shot count.

## Fluid balance and hemodynamic stabilization

Fluid resuscitation represents a cornerstone in supportive therapy of septic patients. However, the 'what, when and how' of the treatment is currently discussed controversially. The Surviving Sepsis Campaign Guidelines 2008 recommend fast initial fluid resuscitation and hemodynamic stabilization within 6 hours [1]. This recommendation follows from the study by Rivers and colleagues, who performed a randomized controlled clinical trial on 263 emergency care patients with severe sepsis or septic shock. Patients were randomized to receive either 6 hours of standard therapy or 6 hours of central venous oxygenation-guided EGDT before admission to the ICU [6]. Since in-house mortality was 30.5% in the EGDT group and 46.5% in the standard therapy group (*P *= 0.009), the authors concluded that EGDT provides significant benefits for patients with sepsis and septic shock.

A retrospective pilot study by Alsous and colleagues investigated the impact of achieving a negative fluid balance (≥500 ml) on at least 1 day of the first 3 days of treatment in 36 septic shock patients. Within this trial, all patients with at least 1 day of negative fluid balance survived (*n *= 11). The authors therefore concluded that a negative fluid balance early in the course of the treatment predicts survival [7]. Another study evaluating the effects of fluid balance on mortality was performed by Boyd and colleagues in a *post hoc *analysis of the Vasopressin and Septic Shock Trial. The fluid balance of 778 patients was analyzed on the first 4 days of treatment and divided into four quartiles, where 1 represents patients with the most positive fluid balance and 4 those patients with the least positive fluid balance. The data clearly show that a positive fluid balance early in the treatment (after 12 hours) and cumulatively (on day 4) was associated with increased mortality (each *P *<0.05) [8].

Based on the available literature, early hemodynamic stabilization seems to be beneficial for septic patients. However, a positive fluid balance may potentially worsen patient outcome. From a rational (physiological) point of view, it makes sense to infuse liberal amounts of fluids in the initial state of hemodynamic instability. When the patient is stabilized (for example, needs no vasopressor support any longer), it appears useful to target a negative fluid balance. As stated by Dr Rivers, 'early liberal, late conservative' might be the way to go [9]. Another important aspect is that fluid resuscitation should be appropriate and demand-oriented. General attempts to keep the patient wet or dry should therefore be revisited [10]. A key remaining question is: which (cardiovascular) targets may actually be considered as valid in hemodynamic support?

Although central venous pressure (CVP) is routinely used to guide fluid therapy, several studies have provided evidence that CVP represents an un reliable variable in this context [8,11]. Broch and colleagues performed a clinical trial with 92 patients undergoing coronary artery surgery with the aim of finding the ideal predictor of fluid responsiveness [12]. The global end-diastolic volume index and respiratory variations in left ventricular outflow tract velocity were compared with pulse pressure variation and stroke volume variation. Responding was defined as an increase in stroke volume index >15% during passive leg raising. Whereas CVP was not able to predict fluid responsiveness and showed no correlation with the stroke volume index, the global end-diastolic volume index and respiratory variations in left ventricular outflow tract velocity turned out to be reliable predictors of fluid responsiveness. Furthermore, pulse pressure variation and stroke volume variation showed the highest accuracy in predicting an increase in the stroke volume index [12].

Taken together, early demand-oriented and appropriate hemodynamic stabilization in septic patients is desirable. However, potential harmful effects of subsequent fluid overload should be taken into consideration. Although large prospective outcome studies are still lacking, the available literature suggests that dynamic hemodynamic parameters are superior to CVP in predicting fluid responsiveness in hemodynamically unstable patients.

## Sedation in the ICU

To facilitate mechanical ventilation in the ICU, sedative agents are often used (in large amounts) [13,14]. Although specific protocols for sedation and mechanical ventilation may reduce ICU length of stay and improve outcome [14], it has recently been reported that a daily interruption of sedation (wake-up call) did not reduce the duration of mechanical ventilation and ICU length of stay, but increased the overall need for benzodiazepines and the workload of the nurses [15].

The most widely used sedatives in Europe are propofol and midazolam, often combined with opioids [16]. A clinical trial in 60 patients showed that a combination of haloperidol and propofol reduced the occurrence of respiratory depression when compared with midazolam - propofol [17].

The role of dexmedetomidine for sedation of ICU patients was evaluated recently in two randomized controlled trials. The investigators reported that the latter agent shortened the duration of mechanical ventilation versus midazolam but was associated with more adverse effects [18].

With respect to long-term sedation, Theilen and colleagues compared the pharmacological characteristics of propofol in medium-chain and long-chain triglyceride emulsion. Thirty patients who required mechanical ventilation for at least 48 hours received either propofol 2% medium-chain triglyceride/long-chain triglyceride or propofol 2% long-chain triglyceride, followed by measurements concerning propofol serum and plasma triglyceride levels. Interestingly, the medium-chain tryglyceride/long-chain triglyceride group was characterized by a faster elimination of the triglycerides post treatment [19]. Another trial by Mesnil and colleagues compared the effects of inhaled sevoflurane with intravenous propofol and midazolam in 47 patients receiving sedation for at least 24 hours. The primary endpoints were wake-up times and extubation delay after termination of sedation. The patients allocated to the sevoflurane group showed significantly shorter wake-up time and extubation delay (*P *<0.01) as compared with the intravenous groups. Similarly, the morphine consumption was lower, and no hallucination episodes occurred during the 24-hour post-extubation period in the sevoflurane group [20]. The use of volatile agents in the ICU, however, requires a specialist medical device and trained staff.

While experts argue what might be the best sedative agent, a Scandinavian team led by Strøm performed a single-blinded cohort study in which 140 mechanically ventilated patients were randomly assigned to receive either sedation with daily wake up or no sedation [21]. Two years later, 13 patients from each group were interviewed by a neuropsychologist concerning quality of life, the Becks depression index and state anxiety scores. Since there were no differences between the two groups, the authors concluded that a no-sedation protocol does not increase the risk of psychological sequelae when compared with a standard sedation protocol [21].

In addition to the choice of the right sedative agent in mechanically ventilated patients, the correct timing of and protocols for the weaning assessment are also of crucial importance. In this context, Girard and colleagues investigated the effects of two different weaning protocols in 336 ventilated patients. The primary endpoint was spontaneous breathing without assistance. The patients were randomly assigned to undergo daily spontaneous awakening trials followed by spontaneous breathing trials or sedation plus daily spontaneous breathing trials. Interestingly, patients in the intervention group (spontaneous awakening trials + spontaneous breathing trials) were characterized by more days of breathing without assistance, earlier discharge from the ICU and hospital, as well as a lower mortality in the year after randomization as compared with the control group (each *P *<0.05) [22].

When reviewing the current literature on this topic, it appears that the concept of 'as much as needed and the least possible' is most appropriate to enable a fast extubation and to prevent iatrogenic arterial hypotension secondary to the use of (high doses of) vasodilatory agents.

## Renal replacement therapy in the ICU

While more than 25% of critically ill patients in the ICU develop acute kidney injury, there are two contrary opinions about how to deal with the renal replacement therapy (RRT). Rimes-Stigare and colleagues reviewed 22 studies concerning adults with acute kidney injury and RRT. According to the authors, early initiation of RRT and fine-tuning of the technique may improve outcome [23]. On the other hand, a study by Elseviers and colleagues in nine ICUs with 1,303 patients (serum creatinine >2 mg/dl) compared conservative treatment (control of volume, electrolytes, and acid-base balance, as well as specific drug management) with either intermittent RRT or continuous RRT. Within this trial, the RRT group showed a higher mortality and prolonged length of ICU and hospital stay - leading to the conclusion that a more critical approach for the use of RRT might be necessary [24].

Is it now timely to ask whether or not we should use RRT in the ICU? Perhaps it is less a question of 'yes or no', but rather about 'when and how' to use RRT.

Regarding the 'how', the VA/NIH Acute Renal Failure Trial Network performed a randomized controlled trial with 1,124 critically ill patients suffering from acute kidney injury and failure of at least one non-renal organ or sepsis [25]. The patients were randomly assigned to receive either intensive RRT (defined as intermittent hemodialysis and low-efficiency dialysis six times per week and continuous venovenous hemodialysis of 35 ml/kg/hour) or less intensive RRT (defined as corresponding treatments three times per week with 20 ml/kg/hour). The primary endpoint was death by any cause 60 days post randomization. Interestingly, no significant difference was found between the treatment groups regarding mortality, recovery of kidney function and duration of RRT [25]. One year later, a similar multicenter randomized controlled trial with 1,508 critically ill adults suffering from acute kidney injury was performed by Bellomo and colleagues. The patients underwent either higher intensity RRT (continuous venovenous hemodialysis, effluent flow 40 ml/kg/hour) or lower intensity RRT (continuous venovenous hemodialysis, effluent flow 25 ml/kg/hour). While there were no significant differences between the treatment groups concerning 90-day mortality (44.7% in each group), the patients of the intensive RRT group had a higher rate of hypophosphatemia (*P *<0.001) [26].

According to these two large-scale studies, there seems to be no obvious benefit in 'hit-hard' treatment concerning RRT, which should enable us to reflect on whether 'hit medium' might be enough. However, the right time point for RRT initiation remains to be clarified.

A large meta-analysis by Karvellas and colleagues compared the effects of 'early versus late initiation of RRT in critically ill patients with acute kidney injury' [27]. In this systematic review of 15 original studies (two randomized, four prospective, nine retrospective cohort), early initiation of RRT showed a significant improvement in 28-day survival as compared with late initiation of RRT (odds ratio = 0.45, 95% confidence interval = 0.28 to 0.72). For completeness, however, one should note that the overall results were mostly derived from small trials with differences in quality and design. Furthermore, the criteria for early and late initiation differed between the reviewed studies, implying that the authors' conclusion about the beneficial effects of early RRT should be handled with care. While there is no evidence-based recommendation regarding when RRT should be initiated, initiation of RRT remains a non-standardized, subjective decision.

Summarizing the current evidence, low-intensive continuous RRT (20 to 30 ml/kg/hour) or intermittent RRT three times weekly is suggested, whereas continuous RRT should be the first choice for hemodynamically instable patients [28]. The recommendations concerning the right timing still remain inhomogeneous. Early RRT seems to be beneficial only in the presence of specific comorbidities (that is, fluid overload, sepsis, respiratory failure, and so forth). Further studies are urgently needed to determine the optimal time point for the initiation of RRT. Perhaps new renal biomarkers will help to make initiation of RRT less subjective than it is today.

## Conclusion

Time is relative, even for the critically ill ICU patient. Einstein was right that not all medical interventions have to follow the same rules regarding the timing of their application and removal. Obviously, it is good getting to the point, provided one knows what that point is. In this context, the Tarragona strategy with the implication to 'hit hard and early' has a firm *raison d'être*, especially when the antibiotic is appropriate for the underlying disease. However, this concept should not be uncritically transferred to other medical interventions.

Perhaps Cummings, Franklin and Einstein were all right, so that each of the three theories could have its place in modern medicine. However, with all due respect for these three geniuses, one should always take into consideration that one size does not fit all. All interventions in critically ill patients should therefore be evaluated carefully and individually. Although timing plays a crucial role in the treatment of intensive care patients, 'There is more to life than simply increasing its speed' (Mahatma Gandhi).

## Abbreviations

CVP: central venous pressure; EGDT: early goal-directed therapy; RRT: renal replacement therapy.

## Competing interests

The author declares that they have no competing interests.

## Declarations

This article has been published as part of *Critical Care *Volume 17 Suppl 1, 2013: Future of Critical Care Medicine. The supplement was proposed by Fresenius Kabi based on presentations from the 'Future of critical care medicine (FCCM) 2012: Today's practice and a look to the future' symposium. Articles were commissioned by the journal, were independently prepared by the authors and have been peer reviewed by the journal. The supplement was edited by John Marini, who declares that he has no competing interests. Publication of the supplement was supported by Fresenius Kabi.

## References

[B1] Dellinger RP Levy MM Carlet JM Bion J Parker MM Jaeschke R Reinhart K Angus DC Brun-Buisson C Beale R Calandra T Dhainaut JF Gerlach H Harvey M Marini JJ Marshall J Ranieri M Ramsay G Sevransky J Thompson BT Townsend S Vender JS Zimmerman JL Vincent JL International Surviving Sepsis Campaign Guidelines Committee; American Association of Critical-Care Nurses; American College of Chest Physicians; American College of Emergency Physicians; Canadian Critical Care Society; European Society of Clinical Microbiology and Infectious Diseases Surviving Sepsis Campaign: international guidelines for management of severe sepsis and septic shock: 2008 Crit Care Med 2008 17 296 32718158437

[B2] Bodi M Ardanuy C Olona M Castander D Diaz E Rello J Therapy of ventilator-associated pneumonia: the Tarragona strategy Clin Microbiol Infect 2001 17 32 33 11284942 10.1046/j.1469-0691.2001.00187.x

[B3] Kumar A Roberts D Wood KE Light B Parrillo JE Sharma S Suppes R Feinstein D Zanotti S Taiberg L Gurka D Kumar A Cheang M Duration of hypotension before initiation of effective antimicrobial therapy is the critical determinant of survival in human septic shock Crit Care Med 2006 17 1589 1596 16625125 10.1097/01.CCM.0000217961.75225.E9

[B4] Gaieski DF Mikkelsen ME Band RA Pines JM Massone R Furia FF Shofer FS Goyal M Impact of time to antibiotics on survival in patients with severe sepsis or septic shock in whom early goal-directed therapy was initiated in the emergency department Crit Care Med 2010 17 1045 1053 20048677 10.1097/CCM.0b013e3181cc4824

[B5] Puskarich MA Trzeciak S Shapiro NI Arnold RC Horton JM Studnek JR Kline JA Jones AE Association between timing of antibiotic administration and mortality from septic shock in patients treated with a quantitative resuscitation protocol Crit Care Med 2011 17 2066 2071 21572327 10.1097/CCM.0b013e31821e87ab PMC3158284

[B6] Rivers E Nguyen B Havstad S Ressler J Muzzin A Knoblich B Peterson E Tomlanovich M Early goal-directed therapy in the treatment of severe sepsis and septic shock N Engl J Med 2001 17 1368 1377 11794169 10.1056/NEJMoa010307

[B7] Alsous F Khamiees M DeGirolamo A Amoateng-Adjepong Y Manthous CA Negative fluid balance predicts survival in patients with septic shock: a retrospective pilot study Chest 2000 17 1749 1754 10858412 10.1378/chest.117.6.1749

[B8] Boyd JH Forbes J Nakada TA Walley KR Russell JA Fluid resuscitation in septic shock: a positive fluid balance and elevated central venous pressure are associated with increased mortality Crit Care Med 2011 17 259 265 20975548 10.1097/CCM.0b013e3181feeb15

[B9] Rivers EP Fluid-management strategies in acute lung injury - liberal, conservative, or both? N Engl J Med 2006 17 2598 2600 16714769 10.1056/NEJMe068105

[B10] Westphal M Scholz J Van Aken H Bein B Infusion therapy in anaesthesia and intensive care: let's stop talking about 'wet' and 'dry'! Best Pract Res Clin Anaesthesiol 2009 17 vii x 19653434 10.1016/j.bpa.2009.04.001

[B11] Brisman R Parks LC Benson DW Pitfalls in the clinical use of central venous pressure Arch Surg 1967 17 902 907 6058794 10.1001/archsurg.1967.01330180050009

[B12] Broch O Renner J Gruenewald M Meybohm P Hocker J Schottler J Steinfath M Bein B Variation of left ventricular outflow tract velocity and global end-diastolic volume index reliably predict fluid responsiveness in cardiac surgery patients J Crit Care 2012 17 e7 e13 21855280 10.1016/j.jcrc.2011.07.073

[B13] Ely EW Baker AM Dunagan DP Burke HL Smith AC Kelly PT Johnson MM Browder RW Bowton DL Haponik EF Effect on the duration of mechanical ventilation of identifying patients capable of breathing spontaneously N Engl J Med 1996 17 1864 1869 8948561 10.1056/NEJM199612193352502

[B14] Kress JP Pohlman AS O'Connor MF Hall JB Daily interruption of sedative infusions in critically ill patients undergoing mechanical ventilation N Engl J Med 2000 17 1471 1477 10816184 10.1056/NEJM200005183422002

[B15] Mehta S Early sedation of mechanically ventilated, critically ill patients: another wake-up call! Am J Respir Crit Care Med 2012 17 699 23071182 10.1164/rccm.201208-1387ED

[B16] Soliman HM Melot C Vincent JL Sedative and analgesic practice in the intensive care unit: the results of a European survey Br J Anaesth 2001 17 186 192 11493487 10.1093/bja/87.2.186

[B17] Etezadi F Yarandi KK Moharari RS Khajavi MR ICU sedation with haloperidol-propofol infusion versus midazolam-propofol infusion after coronary artery bypass graft surgery: a prospective, double-blind randomized study Ann Card Anaesth 2012 17 185 189 22772512 10.4103/0971-9784.97974

[B18] Jakob SM Ruokonen E Grounds RM Sarapohja T Garratt C Pocock SJ Bratty JR Takala J Dexmedetomidine vs midazolam or propofol for sedation during prolonged mechanical ventilation: two randomized controlled trials JAMA 2012 17 1151 1160 22436955 10.1001/jama.2012.304

[B19] Theilen HJ Adam S Albrecht MD Ragaller M Propofol in a medium- and long-chain triglyceride emulsion: pharmacological characteristics and potential beneficial effects Anesth Analg 2002 17 923 929 12351269 10.1097/00000539-200210000-00024

[B20] Mesnil M Capdevila X Bringuier S Trine PO Falquet Y Charbit J Roustan JP Chanques G Jaber S Long-term sedation in intensive care unit: a randomized comparison between inhaled sevoflurane and intravenous propofol or midazolam Intensive Care Med 2011 17 933 941 21445642 10.1007/s00134-011-2187-3

[B21] Strøm T Stylsvig M Toft P Long-term psychological effects of a no-sedation protocol in critically ill patients Crit Care 2011 17 R293 22166673 10.1186/cc10586 PMC3388632

[B22] Girard TD Kress JP Fuchs BD Thomason JW Schweickert WD Pun BT Taichman DB Dunn JG Pohlman AS Kinniry PA Jackson JC Canonico AE Light RW Shintani AK Thompson JL Gordon SM Hall JB Dittus RS Bernard GR Ely EW Efficacy and safety of a paired sedation and ventilator weaning protocol for mechanically ventilated patients in intensive care (Awakening and Breathing Controlled trial): a randomised controlled trial Lancet 2008 17 126 134 18191684 10.1016/S0140-6736(08)60105-1

[B23] Rimes-Stigare C Awad A Martensson J Martling CR Bell M Long-term outcome after acute renal replacement therapy: a narrative review Acta Anaesthesiol Scand 2012 17 138 146 22092145 10.1111/j.1399-6576.2011.02567.x

[B24] Elseviers MM Lins RL Van der Niepen P Hoste E Malbrain ML Damas P Devriendt J Renal replacement therapy is an independent risk factor for mortality in critically ill patients with acute kidney injury Crit Care 2010 17 R221 21122146 10.1186/cc9355 PMC3219996

[B25] Palevsky PM Zhang JH O'Connor TZ Chertow GM Crowley ST Choudhury D Finkel K Kellum JA Paganini E Schein RM Smith MW Swanson KM Thompson BT Vijayan A Watnick S Star RA Peduzzi P Intensity of renal support in critically ill patients with acute kidney injury N Engl J Med 2008 17 7 20 18492867 10.1056/NEJMoa0802639 PMC2574780

[B26] Bellomo R Cass A Cole L Finfer S Gallagher M Lo S McArthur C McGuinness S Myburgh J Norton R Scheinkestel C Su S Intensity of continuous renal-replacement therapy in critically ill patients N Engl J Med 2009 17 1627 1638 19846848 10.1056/NEJMoa0902413

[B27] Karvellas CJ Farhat MR Sajjad I Mogensen SS Leung AA Wald R Bagshaw SM A comparison of early versus late initiation of renal replacement therapy in critically ill patients with acute kidney injury: a systematic review and meta-analysis Crit Care 2011 17 R72 21352532 10.1186/cc10061 PMC3222005

[B28] Ricci Z Ronco C Timing, dose and mode of dialysis in acute kidney injury Curr Opin Crit Care 2011 17 556 561 22027405 10.1097/MCC.0b013e32834cd360

